# Focus on the management of thunderclap headache: from nosography to treatment

**DOI:** 10.1007/s10194-011-0302-z

**Published:** 2011-02-18

**Authors:** E. Ferrante, Cristina Tassorelli, P. Rossi, C. Lisotto, G. Nappi

**Affiliations:** 1 Headache Centre, Neurosciences Department, Niguarda Ca’ Granda Hospital, Milan, Italy; 2 Headache Science Centre, IRCCS “C. Mondino National Neurological Institute” Foundation and University Consortium Adaptive Disorders and Head Pain (UCADH), University of Pavia, Pavia, Italy; 3 Headache Clinic INI Grottaferrata, Rome, Italy; 4 Headache Centre, Department of Neuroscience, S. Vito al Tagliamento Hospital, San Vito al Tagliamento (PN), Italy; 5 Chair of Neurology, Sapienza University of Rome, Rome, Italy

**Keywords:** Headache, Thunderclap headache, Nosography, Treatment, Subarachnoid haemorrhage, Reversible cerebral vasocostriction syndrome

## Abstract

Thunderclap headache (TCH) is an excruciating headache characterized by a very sudden onset. Recognition and accurate diagnosis of TCH are important in order to rule out the various, serious underlying brain disorders that, in a high percentage of cases, are the real cause of the headache. Primary TCH, which may recur intermittently and generally has a spontaneous, benign evolution, can thus be diagnosed only when all other potential underlying causes have been excluded through accurate diagnostic work up. In this review, we focus on the management of TCH, paying particular attention to the diagnostic work up and treatment of the condition.

## The nosography of TCH

Fisher [1] used the expression “crash migraines” to describe acute, high-intensity headaches similar to those caused by ruptured saccular aneurysms, but with normal lumbar puncture and angiography. Day and Raskin [2] coined the term “thunderclap headache” to refer to a similar type of headache occurring in a patient who had three severe, acute episodes of headache within 1 week. The brain CT scan and the cerebrospinal fluid (CSF) examination were normal in this patient, while cerebral angiography showed diffuse, multifocal, and segmental cerebral vasospasm with a saccular aneurysm at the origin of the right posterior cerebral artery. No evidence of haemorrhage was found during surgical removal of the aneurysm. The authors concluded that unruptured intracranial aneurysms can present with “thunderclap headache” and that angiography is necessary in patients presenting “headache episodes with the thunderclap profile”, even when CT and CSF are normal.

Thunderclap headache (TCH) is a severe and headache of the explosive type that appears suddenly, like a “clap of thunder”, with peak intensity of the pain occurring at onset (usually within 30 s).

The International Classification of Headache Disorders, second edition (ICHD-II, 2004) categorizes headaches into primary and secondary forms based on the absence or presence of intracranial lesions. This classification has included TCH in Group IV—other primary headaches (code 4.6) (Table 1) and has proposed precise diagnostic criteria [3].

**Table 1:** 

4. Other primary headaches
4.1 Primary stabbing headache
4.2 Primary cough headache
4.3 Primary exertional headache
4.4 Primary headache associated with sexual activity
4.4.1 Preorgasmic headache
4.4.2 Orgasmic headache
4.5 Hypnic headache
4.6 Primary thunderclap headache
4.7 Hemicrania continua
4.8 New daily-persistent headache (NDPH)

The nosography of most headaches included in group IV is not immediate. Although some of them are simply identified according to their trigger—i.e. cough headache, sexual headache, exertional headache—it is mandatory to accurately rule out any possible organic cause (that is to exclude a possible secondary origin) when a patient with one of these provoked headaches is seen for the first time. Indeed a recent publication [4] shows that 18 of 30 patients with sexual headaches with an abrupt and severe headache—which was similar to TCH in most cases—had reversible cerebral vasoconstriction syndrome (RCVS) (code 6.7.3 in ICHD-II).

Thunderclap headache is a rare type of headache with an incidence of 43 cases per 100,000 adults per year [4]. Primary TCH closely mimics secondary forms of TCH and therefore appropriate instrumental investigations are absolutely mandatory to rule out possible organic causes [5]. The close similarity between primary and secondary TCH has led to the hypothesis that the primary form may, in some cases, actually be a wrong diagnosis resulting from the incapacity of instrumental diagnostics to identify the organic cause [6]; for this reason, the alternative term “thunderclap headache of undefined origin” has also been proposed [7].

Some authors [8] showed that patients with recurrent TCH can be divided in two groups based on the result of MRA: those with diffuse vasospasm and those without any (visible) vasospasm. However, both groups had similar clinical features and both showed the same rate of ischaemic complications [6–7% of posterior reversible encephalopathy syndromes (PRES) and of ischaemic stroke]. These authors therefore suggested that “primary TCH” and RCVS may be part of the same spectrum. Ducros et al. [9] showed that 21% of 67 patients with a proven RCVS had an initial normal MRA and they would have been classified as “primary TCH” in the absence of a second MRA repeated after a few days later or of a conventional angiogram. Furthermore, other authors showed that the arterial abnormalities of RCVS, as assessed by non invasive tools, increase several days after TCH onset. Indeed mean flow velocities of middle cerebral artery (MCA) measured by transcranial Doppler are maximal at 18–25 days after TCH onset [10], and MRA vasoconstriction scores are maximal 16 ± 10 days after headache onset, close to headache resolution at 16 ± 9 days [11].

Therefore, it is possible that some of “primary” TCHs are actually forms of RCVS whose arterial abnormalities went undetected by neuroimaging. The clinical and diagnostic overlapping is also reflected in the pathophysiological field where the proposed mechanisms for the so-called primary TCH are vasospasm and autonomic dysfunction, which are also thought to underline RCVS. A certain degree of overlapping between TCH and RCVS also exists as regards treatment because nimodipine shows beneficial effect both in the presence and in the absence of vasospasm. RCVS is a vascular disorder that is frequently, but by no means always, benign. Stroke occurs in 5–15% of the prospective series [4, 9] and death has also been reported. Thus, the clinical spectrum of RCVS is large and it ranges from isolated recurrent and self-limiting TCH to life-threatening forms. Severe forms of RCVS were previously misdiagnosed as “benign” forms of primary angiitis of the central nervous system (PACNS), with good evolution and prognosis, and normalization of arterial irregularities after a short course of steroids. Calabrese et al. [12] first proposed that these patients had benign angiitis of the central nervous system (BACNS) and later on recognized that BACNS was equivalent to RCVS.

True primary TCH has a relatively benign prognosis. Although headache can recur within the first week after onset, it generally does not recur regularly over subsequent weeks or months [3]. As regards the pathophysiology of primary TCH, the sympathetic nervous system probably plays an important role. The proximal portions of the large intracranial arteries are indeed innervated by neuropeptide Y and noradrenaline-containing sympathetic afferents, which modulate vascular tone [13, 14]. It has been suggested that the head pain in TCH may be due to acute vasoconstriction or alterations in vascular tone secondary to heightened sympathetic tone, and indeed there exist experimental and clinical data supporting a pivotal role of the sympathetic nervous system (e.g. TCH attacks associated with hypertension or preceded by events associated with elevated sympathetic tone, such as episodes of anger, sexual intercourse, and exertion) [15, 16]. Vasoconstriction per se does not seem to be the cause of pain, since in the cases of TCH with RCVS it is a long-lasting phenomenon, which may persist for hours up or weeks, even when headache has already disappeared [17]. It seems more likely that pain in TCH results from a combination of vasoconstriction with systemic or local autonomic changes. In patients with sexual headache resembling TCH associated with RCVS, additional factors that may contribute to pain induction are possibly represented by the psychological stress associated with sexual arousal and by the activation of systemic autonomic reflexes, which result in increased blood pressure and heart rate [18].

As regards the pathophysiology of the arterial spasm, several factors—encompassing mechanical stimulation, biochemical mediators, and neurogenic events—may be invoked. Vasoactive substances, i.e. ergotamine, amphetamine, cocaine may trigger it. Alternatively, the formation of circulating metabolites (i.e. during eclampsia or pheochromocytoma) or the exposure to toxins (i.e. angiographic contrast material) are all possible causes for vasospasm [19].

## The diagnostic work-up

It is essential to remember that a diagnosis of primary or secondary TCH can be made only when exhaustive instrumental investigations have been performed. To manage a patient with TCH correctly, the clinician must be aware of all the organic disorders that can act as a causative factors. These are, first of all, subarachnoid haemorrhage (SAH), but also cerebral venous sinus thrombosis, carotid artery dissection, hypertensive encephalopathy, spontaneous retroclival haematoma, sentinel headache, ischaemic stroke, pituitary apoplexy, spontaneous intracranial hypotension, colloid cyst of the third ventricle, intracranial infection, PACNS. It is also worth noting that a headache with clinical features of TCH has been described in conditions not associated with a structural abnormality, such as bathing headache, primary cough, sexual and exertional headaches, and RCVS. Recently, some authors have proposed diagnostic criteria for TCH attributed to idiopathic reversible cerebral vasoconstriction (THARCV) syndrome based on the clinical and radiological features [18].

Table 2 shows the diagnostic work up and the main criteria for the differential diagnosis of primary TCH versus other disorders that can cause/be associated with TCHs [12–88].

**Table 2:** Differential diagnosis of TCH

Disease	Neurological symptoms/signs	Precipitating factors	Comments
Subarachnoid haemorrhage (SAH) [19–32]	Headache	Physical exertion	About 70% of pts with SAH present with headache alone
	Loss of consciousness	Sexual intercourse	
	Focal neurological symptoms		
Cerebral venous sinus thrombosis (CVST) [33–42]	Headache	Puerperium	15–30% of pts present with isolated headache that can worsen in the recumbent position
	Altered consciousness	Dehydration	
	Focal neurological symptoms/signs	Cancer	
Cervical artery dissection (CAD) [3, 43–45]	Headache	Head and neck injury	Generally headache is ipsilateral to the CAD
	Amaurosis fugax		
	Horner’s syndrome		
	Focal neurological symptoms/signs		
Acute hypertensive crisis (AHC) [46–51]	Headache	Hypertensive crisis	Headache occurs in about 20% of pts with AHC
	Altered mental status		
	Seizures		
	Focal neurological symptoms/signs		
Spontaneous retroclival hematoma (SRH) [52, 53]	Headache	None	SRH is very rare
	Mild nuchal rigidity		
	Oculomotor nerve palsy		
	Upper limb paresis		
Sentinel headache (SH) [54]	Headache	Physical exertion	SH is present in 10–40% of pts with SAH
	Focal neurological symptoms/signs generally absent	Sexual intercourse	
Ischaemic stroke (IS) [55–58]	Headache	None	Headache is more common with large IS
	Focal neurological symptoms/signs		
Pituitary apoplexy (PA) [22, 58–60]	Headache	Pregnancy	PA commonly occurs in pts with no known pituitary tumour history
	Visual disturbances		
Spontaneous intracranial hypotension (SIH) [61–64]	Orthostatic headache	Valsalva manoeuvre	TCH is present, at onset, in about 15% of pts with SIH
	Hearing disturbances		
	Mild nuchal rigidity		
Colloid cysts of third ventricle [73–75]	Headache	None	Headache can be relieved by recumbency
	Loss of consciousness		
	Seizures		
	Coma		
Reversible cerebral vasoconstriction syndrome (RCVS) [8, 69–73]	Headache	Postpartum	RCVS is spontaneous in about 30% of cases
	Focal neurological symptoms/signs	Sexual intercourse	
		Drugs exposure (see bottom of table)	Prognosis is uncertain, but most pts do well
		Blood products (see bottom of table)	
		Head trauma	
		Neurosurgical procedures	
Benign hot bath-related headache [74–78]	Headache	Hot bath	Headache disappears spontaneously after a period of 2 weeks to 3 months
	Normal neurological examination	Hot shower	
Primary cough,	Headache	Cough	These headache forms are
sexual and exertional headaches [3, 79]	Normal neurological examination	Physical exertion	an exclusion diagnosis
		Sexual activity	
Primary angiitis of the central nervous system (PACNS) [72, 80–83]	Headache	None	Headache is the most common presenting symptom
	Seizures		
	Behavioural disturbances		
	Focal neurological symptoms/signs		
Primary thunderclap headache (TCH) [3, 12–15, 24, 26, 84–89]	Headache	None	TCH is an exclusion diagnosis and has a relatively benign prognosis
	Normal neurological examination		

*Drugs exposure* phenylpropanolamine, ergotamine tartrate, methergine, bromocryptine, lisuride, tricyclic antidepressants, selective serotonin reuptake inhibitors, sumatriptan, isometheptine, cocaine, ecstasy, amphetamine derivatives, marijuana, lysergic acid diethylamide, tacrolimus (FK-506), cyclophosphamide

*Blood products* erythropoietin, intravenous immune globulin, and red blood cell transfusions

While defining the diagnosis, TCH must always be managed as a medical emergency in order to avoid potentially catastrophic consequences that can occur with secondary TCH. The initial diagnostic assessment must be aimed at ruling out SAH. Non-contrast brain CT is the first examination in this assessment, to be performed within the first 12 h after the onset of the headache, preferably using third-generation CT scanners that have a specificity of 98% and a sensitivity close to 100% [25]. The sensitivity of CT for the detection of SAH declines with increasing time from haemorrhage onset, falling to 86% on day one, 76% after 2 days, and 58% after 5 days [84]. Therefore, although the sensitivity of CT scan in detecting SAH is very high in the early phase, if we consider also the possibility of human error, which, in the case of SAH, may be fatal, we recommend to perform lumbar puncture even in the presence of a normal brain CT. CSF assessment is definitely mandatory in patients who come to clinical attention 12 h after TCH onset and have normal or non-diagnostic brain CT scans. Blood and CSF work up (routine cell counts, measurement of protein, glucose, opening pressure, and inspection for xanthochromia) should be performed. Because visual inspection for xanthochromia is associated with a high rate of false-negative interpretations, spectrophotometry should be performed when available. Spectrophotometry also helps to overcome the problem of false-positives; analysis for bilirubin by spectrophotometry has a sensitivity close to 100% when lumbar puncture is performed between 12 h and 2 weeks after SAH [27].

Magnetic resonance imaging (MRI), which can detect many of the possible causes of secondary TCH, should be performed in all TCH patients with normal or non-diagnostic CT scans and CSF analysis. In most cases, magnetic resonance studies should include cerebral MRI, magnetic resonance angiography, magnetic resonance venography and, if necessary, MRI of the cervical arteries using the fat saturation technique. CT angiography can be used instead of magnetic resonance angiography for the diagnosis of an intracranial aneurysm. Evidence suggests that conventional cerebral angiography is not necessary in the assessment of patients with TCH, normal neurological examinations, and normal CT and lumbar puncture [32].

Some authors believe that conventional cerebral angiography should be avoided, since the contrast medium could enhance the vasospasm and this, in turn, would increase the chance of a stroke or even cause further deterioration of a critical neurological condition [18]. However, since there is no adequate evidence to conclude that conventional angiography can result in worsening vasoconstriction, in highly selected cases, when the clinical suspicion of intracranial aneurysm remains high despite normal or non-diagnostic CT, lumbar puncture, and MRI studies, conventional angiography has to be considered.

## The treatment

The following indications have been derived from the information collected from a systematic analysis of the international literature. We conducted a literature search covering the period 1932–2010, employing available electronic databases (National Library of Medicine, National Institute of Health, Embase) with the following medical search terms: “thunderclap”, “cough”, “exertional”, “exercise”, “orgasmic”, “sex”, or “abrupt” in association with “headache”. Whenever available, chapters of book were also consulted and considered. Unfortunately, the literature contains very few reports on the treatment of primary TCH and those that are available are mostly represented by single case reports. Primary TCH generally responds poorly to analgesics. In one report, a woman with an apparent primary TCH had frequent recurrences of the headache until she reached (by day 14) a therapeutic dosage of gabapentin 600 mg three times a day [85]. The exact mechanism of action through which gabapentin decreases headache pain is not known, although multiple mechanisms might be involved. Gabapentin enhances GABA-mediated inhibition, inhibits GABA metabolism and modulates L-type calcium channels by binding to their α2δ subunit [86].

Nimodipine showed beneficial effect both in the presence and in the absence of vasospasm. IV nimodipine and magnesium were administered to a 63-year-old woman with TCH and vasospasm; a posterior ischaemic infarct had occurred before infusion [87]. The patient’s headaches resolved within hours, and transcranial Doppler sonography revealed normalized mean cerebral blood flow velocity, suggesting relief of the vasospasm. The mean cerebral blood flow velocity increased again when oral nifedipine, another calcium channel blocker, was used in place of nimodipine. Nimodipine infusion effectively stopped TCH in another woman, aged 58 years, who had vasospasm and posterior ischaemic infarct. Serial MR angiography showed marked improvement of vasospasm within 2 days [88].

Lu et al. [89] described 11 patients with primary TCH (nine without vasospasm and two with vasospasm) treated with nimodipine. In eight of the nine patients without vasospasm, TCH stopped within 24 h of oral nimodipine administration; the other patient had one further attack 2 days later. The highest dosage of nimodipine was 30 mg every 4 h in four patients, 45 mg every 4 h in one, and 60 mg every 4 h in four. No hypotension or other adverse effects were noted. The treatment lasted 3–4 weeks with gradual tapering. In the two patients with arterial vasospasm, oral nimodipine was only temporarily effective and TCH recurred. When IV nimodipine was given instead, TCH subsided in 6 h without recurrence. In one of the patients, the dose infused was 2 mg/h; in the other, it was lower (0.5–1 mg/h) because of the appearance of nimodipine-induced hypotension. The IV route was switched to oral when MR angiography or transcranial Doppler sonography no longer showed evidence of vasospasm. The duration of IV nimodipine treatment was variable, ranging from 5 to 10 days. No patient reported a relapse of TCH during a mean 6-month follow-up (range 1–19 months) after nimodipine discontinuation. These reports suggest that IV nimodipine is the drug of choice for primary TCH with cerebral vasospasm, whereas oral nimodipine can be used in patients without vasospasm, although the optimal dose and time window remain to be determined. It is noteworthy that the patients with TCH associated with vasospasm described in the above reports [87–89], the correct diagnosis should be RCVS rather than TCH, while only the nine without vasospasm described by Lu et al. [89] can technically be classified as having primary TCH, although with a certain degree of approximation since one cannot exclude a RCVS without visible vasoconstriction at the time of angiogram. Therefore, these nine subjects can be regarded either as “probable RCVS” or “probable primary TCH”.

Following this line of reasoning, if we consider that, in some cases, RVCS may be missed by MRA because of timing issues, it seems wise to avoid tricyclic antidepressants (e.g. amitriptyline) and propranolol because they may facilitate the development of vasoconstriction as suggested by Valenca et al. [18]. In analogy, vasoconstrictor medications, such as ergots and triptans, should be contraindicate during the treatment of headache of patients with potential cerebral vasoconstriction syndromes, at least in the acute phase and until ongoing or impending RVCS has been ruled out.

## Conclusions

All patients with TCH profiles must be assessed urgently and thoroughly in order to consider and rule out all the possible organic causes. After the exclusion of all secondary causes, including RCVS, there are very few patients left with true “primary TCH”.

The data gathered in recent years suggest that, in order to avoid false primary TCHs, it is appropriate to perform neuroimaging studies (brain MRI angiography or CT angiography) 3–4 weeks after the onset of all cases of TCH with negative instrumental findings during the acute phase in order to exclude the presence of delayed vasospasm [10, 11] and therefore identify the true primary TCHs.

Once the diagnosis has been defined with certainty, secondary forms of TCH must be managed through treatment of the underlying brain disorder. For primary TCH, as well as for forms associated with RCVS, the therapeutic options are restricted to nimodipine, intravenously or orally administered, although gabapentin was reported effective in one case of primary TCH.
